# Supplementary material for: The effects of genital myiasis on the diversity of the vaginal microbiota in female Bactrian camels
Source: BMC Vet Res. 2022 Mar 5;18:87. doi: 10.1186/s12917-022-03189-5 (PMC8897907; doi:10.1186/s12917-022-03189-5)
Supplement: Supplementary file 5 — Additional file 5. [file 12917_2022_3189_MOESM5_ESM.zip › MPL201709200_16s_yy/Treat1/css/js_css/jsPDF-master.zip!/jsPDF-master/docs/plugins_context2d.js.html]

plugins/context2d.js - Documentation


## Home

### Classes

- jsPDF

### Global

- addFont
- addHTML
- addMetadata
- addPage
- autoPrint
- CapJoinStyles
- circle
- ellipse
- getFontList
- lines
- lstext
- output
- rect
- roundedRect
- save
- setDisplayMode
- setDrawColor
- setFillColor
- setFont
- setFontSize
- setFontStyle
- setLineCap
- setLineJoin
- setLineWidth
- setPage
- setProperties
- setTextColor
- text
- triangle

# plugins/context2d.js

```
/**
 * jsPDF Context2D PlugIn Copyright (c) 2014 Steven Spungin (TwelveTone LLC) steven@twelvetone.tv
 *
 * Licensed under the MIT License. http://opensource.org/licenses/mit-license
 */

/**
 * This plugin mimics the HTML5 Canvas's context2d.
 *
 * The goal is to provide a way for current canvas implementations to print directly to a PDF.
 */

/**
 * TODO implement stroke opacity (refactor from fill() method )
 * TODO transform angle and radii parameters
 */

/**
 * require('jspdf.js'); require('lib/css_colors.js');
 */

(function (jsPDFAPI) {
    'use strict';

    jsPDFAPI.events.push([
        'initialized', function () {
            this.context2d.pdf = this;
            this.context2d.internal.pdf = this;
            this.context2d.ctx = new context();
            this.context2d.ctxStack = [];
            this.context2d.path = [];
        }
    ]);

    jsPDFAPI.context2d = {
        pageWrapXEnabled: false,
        pageWrapYEnabled: false,
        pageWrapX: 9999999,
        pageWrapY: 9999999,
        ctx: new context(),
        f2: function (number) {
            return number.toFixed(2);
        },

        fillRect: function (x, y, w, h) {
            if (this._isFillTransparent()) {
                return;
            }
            x = this._wrapX(x);
            y = this._wrapY(y);

            var xRect = this._matrix_map_rect(this.ctx._transform, {x: x, y: y, w: w, h: h});
            this.pdf.rect(xRect.x, xRect.y, xRect.w, xRect.h, "f");
        },

        strokeRect: function (x, y, w, h) {
            if (this._isStrokeTransparent()) {
                return;
            }
            x = this._wrapX(x);
            y = this._wrapY(y);

            var xRect = this._matrix_map_rect(this.ctx._transform, {x: x, y: y, w: w, h: h});
            this.pdf.rect(xRect.x, xRect.y, xRect.w, xRect.h, "s");
        },

        /**
         * We cannot clear PDF commands that were already written to PDF, so we use white instead. <br />
         * As a special case, read a special flag (ignoreClearRect) and do nothing if it is set.
         * This results in all calls to clearRect() to do nothing, and keep the canvas transparent.
         * This flag is stored in the save/restore context and is managed the same way as other drawing states.
         * @param x
         * @param y
         * @param w
         * @param h
         */
        clearRect: function (x, y, w, h) {
            if (this.ctx.ignoreClearRect) {
                return;
            }

            x = this._wrapX(x);
            y = this._wrapY(y);

            var xRect = this._matrix_map_rect(this.ctx._transform, {x: x, y: y, w: w, h: h});
            this.save();
            this.setFillStyle('#ffffff');
            //TODO This is hack to fill with white.
            this.pdf.rect(xRect.x, xRect.y, xRect.w, xRect.h, "f");
            this.restore();
        },

        save: function () {
            this.ctx._fontSize = this.pdf.internal.getFontSize();
            var ctx = new context();
            ctx.copy(this.ctx);
            this.ctxStack.push(this.ctx);
            this.ctx = ctx;
        },

        restore: function () {
            this.ctx = this.ctxStack.pop();
            this.setFillStyle(this.ctx.fillStyle);
            this.setStrokeStyle(this.ctx.strokeStyle);
            this.setFont(this.ctx.font);
            this.pdf.setFontSize(this.ctx._fontSize);
            this.setLineCap(this.ctx.lineCap);
            this.setLineWidth(this.ctx.lineWidth);
            this.setLineJoin(this.ctx.lineJoin);
        },

        rect: function (x, y, w, h) {
            this.moveTo(x, y);
            this.lineTo(x + w, y);
            this.lineTo(x + w, y + h);
            this.lineTo(x, y + h);
            this.lineTo(x, y); //TODO not needed
            this.closePath();
        },

        beginPath: function () {
            this.path = [];
        },

        closePath: function () {
            this.path.push({
                type: 'close'
            });
        },

        _getRgba: function (style) {
            // get the decimal values of r, g, and b;
            var rgba = {};

            if (this.internal.rxTransparent.test(style)) {
                rgba.r = 0;
                rgba.g = 0;
                rgba.b = 0;
                rgba.a = 0;
            }
            else {
                var m = this.internal.rxRgb.exec(style);
                if (m != null) {
                    rgba.r = parseInt(m[1]);
                    rgba.g = parseInt(m[2]);
                    rgba.b = parseInt(m[3]);
                    rgba.a = 1;
                } else {
                    m = this.internal.rxRgba.exec(style);
                    if (m != null) {
                        rgba.r = parseInt(m[1]);
                        rgba.g = parseInt(m[2]);
                        rgba.b = parseInt(m[3]);
                        rgba.a = parseFloat(m[4]);
                    } else {
                        rgba.a = 1;
                        if (style.charAt(0) != '#') {
                            style = CssColors.colorNameToHex(style);
                            if (!style) {
                                style = '#000000';
                            }
                        } else {
                        }

                        if (style.length === 4) {
                            rgba.r = style.substring(1, 2);
                            rgba.r += r;
                            rgba.g = style.substring(2, 3);
                            rgba.g += g;
                            rgba.b = style.substring(3, 4);
                            rgba.b += b;
                        } else {
                            rgba.r = style.substring(1, 3);
                            rgba.g = style.substring(3, 5);
                            rgba.b = style.substring(5, 7);
                        }
                        rgba.r = parseInt(rgba.r, 16);
                        rgba.g = parseInt(rgba.g, 16);
                        rgba.b = parseInt(rgba.b, 16);
                    }
                }
            }
            rgba.style = style;
            return rgba;
        },

        setFillStyle: function (style) {
            // get the decimal values of r, g, and b;
            var r, g, b, a;

            if (this.internal.rxTransparent.test(style)) {
                r = 0;
                g = 0;
                b = 0;
                a = 0;
            }
            else {
                var m = this.internal.rxRgb.exec(style);
                if (m != null) {
                    r = parseInt(m[1]);
                    g = parseInt(m[2]);
                    b = parseInt(m[3]);
                    a = 1;
                } else {
                    m = this.internal.rxRgba.exec(style);
                    if (m != null) {
                        r = parseInt(m[1]);
                        g = parseInt(m[2]);
                        b = parseInt(m[3]);
                        a = parseFloat(m[4]);
                    } else {
                        a = 1;
                        if (style.charAt(0) != '#') {
                            style = CssColors.colorNameToHex(style);
                            if (!style) {
                                style = '#000000';
                            }
                        } else {
                        }

                        if (style.length === 4) {
                            r = style.substring(1, 2);
                            r += r;
                            g = style.substring(2, 3);
                            g += g;
                            b = style.substring(3, 4);
                            b += b;
                        } else {
                            r = style.substring(1, 3);
                            g = style.substring(3, 5);
                            b = style.substring(5, 7);
                        }
                        r = parseInt(r, 16);
                        g = parseInt(g, 16);
                        b = parseInt(b, 16);
                    }
                }
            }

            this.ctx.fillStyle = style;
            this.ctx._isFillTransparent = (a == 0);
            this.ctx._fillOpacity = a;

            this.pdf.setFillColor(r, g, b, {
                a: a
            });
            this.pdf.setTextColor(r, g, b, {
                a: a
            });
        },

        setStrokeStyle: function (style) {
            var rgba = this._getRgba(style);

            this.ctx.strokeStyle = rgba.style;
            this.ctx._isStrokeTransparent = (rgba.a == 0);
            this.ctx._strokeOpacity = rgba.a;

            //TODO jsPDF to handle rgba
            if (rgba.a === 0) {
                this.pdf.setDrawColor(255, 255, 255);
            }
            else if (rgba.a === 1) {
                this.pdf.setDrawColor(rgba.r, rgba.g, rgba.b);
            } else {
                //this.pdf.setDrawColor(rgba.r, rgba.g, rgba.b, {a: rgba.a});
                this.pdf.setDrawColor(rgba.r, rgba.g, rgba.b);
            }
        },

        fillText: function (text, x, y, maxWidth) {
            if (this._isFillTransparent()) {
                return;
            }
            x = this._wrapX(x);
            y = this._wrapY(y);

            var xpt = this._matrix_map_point(this.ctx._transform, [x, y]);
            x = xpt[0];
            y = xpt[1];
            var rads = this._matrix_rotation(this.ctx._transform);
            var degs = rads * 57.2958;


            //TODO only push the clip if it has not been applied to the current PDF context
            if (this.ctx._clip_path.length > 0) {
                var lines;
                if (window.outIntercept) {
                    lines = window.outIntercept.type === 'group' ? window.outIntercept.stream : window.outIntercept;
                } else {
                    lines = this.pdf.internal.pages[1];
                }
                lines.push("q");
                var origPath = this.path;
                this.path = this.ctx._clip_path;
                this.ctx._clip_path = [];
                this._fill(null, true);
                this.ctx._clip_path = this.path;
                this.path = origPath;
            }

            this.pdf.text(text, x, this._getBaseline(y), null, degs);

            if (this.ctx._clip_path.length > 0) {
                lines.push('Q');
            }
        },

        strokeText: function (text, x, y, maxWidth) {
            if (this._isStrokeTransparent()) {
                return;
            }
            x = this._wrapX(x);
            y = this._wrapY(y);

            var xpt = this._matrix_map_point(this.ctx._transform, [x, y]);
            x = xpt[0];
            y = xpt[1];
            var rads = this._matrix_rotation(this.ctx._transform);
            var degs = rads * 57.2958;


            //TODO only push the clip if it has not been applied to the current PDF context
            if (this.ctx._clip_path.length > 0) {
                var lines;
                if (window.outIntercept) {
                    lines = window.outIntercept.type === 'group' ? window.outIntercept.stream : window.outIntercept;
                } else {
                    lines = this.pdf.internal.pages[1];
                }
                lines.push("q");
                var origPath = this.path;
                this.path = this.ctx._clip_path;
                this.ctx._clip_path = [];
                this._fill(null, true);
                this.ctx._clip_path = this.path;
                this.path = origPath;
            }

            this.pdf.text(text, x, this._getBaseline(y), {
                stroke: true
            }, degs);

            if (this.ctx._clip_path.length > 0) {
                lines.push('Q');
            }

        },

        setFont: function (font) {
            this.ctx.font = font;

            //var rx = /\s*(\w+)\s+(\w+)\s+(\w+)\s+([\d\.]+)(px|pt|em)\s+["']?(\w+)['"]?/;
            var rx = /\s*(\w+)\s+(\w+)\s+(\w+)\s+([\d\.]+)(px|pt|em)\s+(.*)?/;
            m = rx.exec(font);
            if (m != null) {
                var fontStyle = m[1];
                var fontVariant = m[2];
                var fontWeight = m[3];
                var fontSize = m[4];
                var fontSizeUnit = m[5];
                var fontFamily = m[6];

                if ('px' === fontSizeUnit) {
                    fontSize = Math.floor(parseFloat(fontSize));
                    // fontSize = fontSize * 1.25;
                } else if ('em' === fontSizeUnit) {
                    fontSize = Math.floor(parseFloat(fontSize) * this.pdf.getFontSize());
                } else {
                    fontSize = Math.floor(parseFloat(fontSize));
                }

                this.pdf.setFontSize(fontSize);

                if (fontWeight === 'bold' || fontWeight === '700') {
                    this.pdf.setFontStyle('bold');
                } else {
                    if (fontStyle === 'italic') {
                        this.pdf.setFontStyle('italic');
                    } else {
                        this.pdf.setFontStyle('normal');
                    }
                }

                var name = fontFamily;
                var parts = name.toLowerCase().split(/\s*,\s*/);
                var jsPdfFontName;

                if (parts.indexOf('arial') != -1) {
                    jsPdfFontName = 'Arial';
                }
                else if (parts.indexOf('verdana') != -1) {
                    jsPdfFontName = 'Verdana';
                }
                else if (parts.indexOf('helvetica') != -1) {
                    jsPdfFontName = 'Helvetica';
                }
                else if (parts.indexOf('sans-serif') != -1) {
                    jsPdfFontName = 'sans-serif';
                }

                else if (parts.indexOf('fixed') != -1) {
                    jsPdfFontName = 'Fixed';
                }
                else if (parts.indexOf('monospace') != -1) {
                    jsPdfFontName = 'Monospace';
                }
                else if (parts.indexOf('terminal') != -1) {
                    jsPdfFontName = 'Terminal';
                }
                else if (parts.indexOf('courier') != -1) {
                    jsPdfFontName = 'Courier';
                }

                else if (parts.indexOf('times') != -1) {
                    jsPdfFontName = 'Times';
                }
                else if (parts.indexOf('cursive') != -1) {
                    jsPdfFontName = 'Cursive';
                }
                else if (parts.indexOf('fantasy') != -1) {
                    jsPdfFontName = 'Fantasy';
                }
                else if (parts.indexOf('serif') != -1) {
                    jsPdfFontName = 'Serif';
                }
                else {
                    jsPdfFontName = 'Serif';
                }

                //TODO check more cases
                var style;
                if ('bold' === fontWeight) {
                    style = 'bold';
                } else {
                    style = 'normal';
                }

                this.pdf.setFont(jsPdfFontName, style);
            } else {
                var rx = /(\d+)(pt|px|em)\s+(\w+)\s*(\w+)?/;
                var m = rx.exec(font);
                if (m != null) {
                    var size = m[1];
                    var unit = m[2];
                    var name = m[3];
                    var style = m[4];
                    if (!style) {
                        style = 'normal';
                    }
                    if ('em' === fontSizeUnit) {
                        size = Math.floor(parseFloat(fontSize) * this.pdf.getFontSize());
                    } else {
                        size = Math.floor(parseFloat(size));
                    }
                    this.pdf.setFontSize(size);
                    this.pdf.setFont(name, style);
                }
            }
        },

        setTextBaseline: function (baseline) {
            this.ctx.textBaseline = baseline;
        },

        getTextBaseline: function () {
            return this.ctx.textBaseline;
        },

        //TODO implement textAlign
        setTextAlign: function (align) {
            this.ctx.textAlign = align;
        },

        getTextAlign: function () {
            return this.ctx.textAlign;
        },

        setLineWidth: function (width) {
            this.ctx.lineWidth = width;
            this.pdf.setLineWidth(width);
        },

        setLineCap: function (style) {
            this.ctx.lineCap = style;
            this.pdf.setLineCap(style);
        },

        setLineJoin: function (style) {
            this.ctx.lineJoin = style;
            this.pdf.setLineJoin(style);
        },

        moveTo: function (x, y) {
            x = this._wrapX(x);
            y = this._wrapY(y);

            var xpt = this._matrix_map_point(this.ctx._transform, [x, y]);
            x = xpt[0];
            y = xpt[1];

            var obj = {
                type: 'mt',
                x: x,
                y: y
            };
            this.path.push(obj);
        },

        _wrapX: function (x) {
            if (this.pageWrapXEnabled) {
                return x % this.pageWrapX;
            } else {
                return x;
            }
        },

        _wrapY: function (y) {
            if (this.pageWrapYEnabled) {
                this._gotoPage(this._page(y));
                return (y - this.lastBreak) % this.pageWrapY;
            } else {
                return y;
            }
        },

        transform: function (a, b, c, d, e, f) {
            //TODO apply to current transformation instead of replacing
            this.ctx._transform = [a, b, c, d, e, f];
        },

        setTransform: function (a, b, c, d, e, f) {
            this.ctx._transform = [a, b, c, d, e, f];
        },

        _getTransform: function () {
            return this.ctx._transform;
        },

        lastBreak: 0,
        // Y Position of page breaks.
        pageBreaks: [],
        // returns: One-based Page Number
        // Should only be used if pageWrapYEnabled is true
        _page: function (y) {
            if (this.pageWrapYEnabled) {
                this.lastBreak = 0;
                var manualBreaks = 0;
                var autoBreaks = 0;
                for (var i = 0; i < this.pageBreaks.length; i++) {
                    if (y >= this.pageBreaks[i]) {
                        manualBreaks++;
                        if (this.lastBreak === 0) {
                            autoBreaks++;
                        }
                        var spaceBetweenLastBreak = this.pageBreaks[i] - this.lastBreak;
                        this.lastBreak = this.pageBreaks[i];
                        var pagesSinceLastBreak = Math.floor(spaceBetweenLastBreak / this.pageWrapY);
                        autoBreaks += pagesSinceLastBreak;
                    }
                }
                if (this.lastBreak === 0) {
                    var pagesSinceLastBreak = Math.floor(y / this.pageWrapY) + 1;
                    autoBreaks += pagesSinceLastBreak;
                }
                return autoBreaks + manualBreaks;
            } else {
                return this.pdf.internal.getCurrentPageInfo().pageNumber;
            }
        },

        _gotoPage: function (pageOneBased) {
            // This is a stub to be overriden if needed
        },

        lineTo: function (x, y) {
            x = this._wrapX(x);
            y = this._wrapY(y);

            var xpt = this._matrix_map_point(this.ctx._transform, [x, y]);
            x = xpt[0];
            y = xpt[1];

            var obj = {
                type: 'lt',
                x: x,
                y: y
            };
            this.path.push(obj);
        },

        bezierCurveTo: function (x1, y1, x2, y2, x, y) {
            x1 = this._wrapX(x1);
            y1 = this._wrapY(y1);
            x2 = this._wrapX(x2);
            y2 = this._wrapY(y2);
            x = this._wrapX(x);
            y = this._wrapY(y);

            var xpt;
            xpt = this._matrix_map_point(this.ctx._transform, [x, y]);
            x = xpt[0];
            y = xpt[1];
            xpt = this._matrix_map_point(this.ctx._transform, [x1, y1]);
            x1 = xpt[0];
            y1 = xpt[1];
            xpt = this._matrix_map_point(this.ctx._transform, [x2, y2]);
            x2 = xpt[0];
            y2 = xpt[1];


            var obj = {
                type: 'bct',
                x1: x1,
                y1: y1,
                x2: x2,
                y2: y2,
                x: x,
                y: y
            };
            this.path.push(obj);
        },

        quadraticCurveTo: function (x1, y1, x, y) {
            x1 = this._wrapX(x1);
            y1 = this._wrapY(y1);
            x = this._wrapX(x);
            y = this._wrapY(y);

            var xpt;
            xpt = this._matrix_map_point(this.ctx._transform, [x, y]);
            x = xpt[0];
            y = xpt[1];
            xpt = this._matrix_map_point(this.ctx._transform, [x1, y1]);
            x1 = xpt[0];
            y1 = xpt[1];

            var obj = {
                type: 'qct',
                x1: x1,
                y1: y1,
                x: x,
                y: y
            };
            this.path.push(obj);
        },

        arc: function (x, y, radius, startAngle, endAngle, anticlockwise) {
            x = this._wrapX(x);
            y = this._wrapY(y);

            var xpt = this._matrix_map_point(this.ctx._transform, [x, y]);
            x = xpt[0];
            y = xpt[1];

            var obj = {
                type: 'arc',
                x: x,
                y: y,
                radius: radius,
                startAngle: startAngle,
                endAngle: endAngle,
                anticlockwise: anticlockwise
            };
            this.path.push(obj);
        },

        drawImage: function (img, x, y, w, h, x2, y2, w2, h2) {
            if (x2 !== undefined) {
                x = x2;
                y = y2;
                w = w2;
                h = h2;
            }
            x = this._wrapX(x);
            y = this._wrapY(y);

            var xRect = this._matrix_map_rect(this.ctx._transform, {x: x, y: y, w: w, h: h});
            var xRect2 = this._matrix_map_rect(this.ctx._transform, {x: x2, y: y2, w: w2, h: h2});

            // TODO implement source clipping and image scaling
            var format;
            var rx = /data:image\/(\w+).*/i;
            var m = rx.exec(img);
            if (m != null) {
                format = m[1];
            } else {
                // format = "jpeg";
                format = "png";
            }

            this.pdf.addImage(img, format, xRect.x, xRect.y, xRect.w, xRect.h);
        },

        /**
         * Multiply the first matrix by the second
         * @param m1
         * @param m2
         * @returns {*[]}
         * @private
         */
        _matrix_multiply: function (m2, m1) {
            var sx = m1[0];
            var shy = m1[1];
            var shx = m1[2];
            var sy = m1[3];
            var tx = m1[4];
            var ty = m1[5];

            var t0 = sx * m2[0] + shy * m2[2];
            var t2 = shx * m2[0] + sy * m2[2];
            var t4 = tx * m2[0] + ty * m2[2] + m2[4];
            shy = sx * m2[1] + shy * m2[3];
            sy = shx * m2[1] + sy * m2[3];
            ty = tx * m2[1] + ty * m2[3] + m2[5];
            sx = t0;
            shx = t2;
            tx = t4;

            return [sx, shy, shx, sy, tx, ty];
        },

        _matrix_rotation: function (m) {
            return Math.atan2(m[2], m[0]);

        },

        _matrix_decompose: function (matrix) {

            var a = matrix[0];
            var b = matrix[1];
            var c = matrix[2];
            var d = matrix[3];

            var scaleX = Math.sqrt(a * a + b * b);
            a /= scaleX;
            b /= scaleX;

            var shear = a * c + b * d;
            c -= a * shear;
            d -= b * shear;

            var scaleY = Math.sqrt(c * c + d * d);
            c /= scaleY;
            d /= scaleY;
            shear /= scaleY;

            if (a * d < b * c) {
                a = -a;
                b = -b;
                shear = -shear;
                scaleX = -scaleX;
            }

            return {
                scale: [scaleX, 0, 0, scaleY, 0, 0],
                translate: [1, 0, 0, 1, matrix[4], matrix[5]],
                rotate: [a, b, -b, a, 0, 0],
                skew: [1, 0, shear, 1, 0, 0]
            };
        },

        _matrix_map_point: function (m1, pt) {
            var sx = m1[0];
            var shy = m1[1];
            var shx = m1[2];
            var sy = m1[3];
            var tx = m1[4];
            var ty = m1[5];

            var px = pt[0];
            var py = pt[1];

            var x = px * sx + py * shx + tx;
            var y = px * shy + py * sy + ty;
            return [x, y];
        },

        _matrix_map_point_obj: function (m1, pt) {
            var xpt = this._matrix_map_point(m1, [pt.x, pt.y]);
            return {x: xpt[0], y: xpt[1]};
        },

        _matrix_map_rect: function (m1, rect) {
            var p1 = this._matrix_map_point(m1, [rect.x, rect.y]);
            var p2 = this._matrix_map_point(m1, [rect.x + rect.w, rect.y + rect.h]);
            return {x: p1[0], y: p1[1], w: p2[0] - p1[0], h: p2[1] - p1[1]};
        },

        _matrix_is_identity: function (m1) {
            if (m1[0] != 1) {
                return false;
            }
            if (m1[1] != 0) {
                return false;
            }
            if (m1[2] != 0) {
                return false;
            }
            if (m1[3] != 1) {
                return false;
            }
            if (m1[4] != 0) {
                return false;
            }
            if (m1[5] != 0) {
                return false;
            }
            return true;
        },

        rotate: function (angle) {
            var matrix = [Math.cos(angle), Math.sin(angle), -Math.sin(angle), Math.cos(angle), 0.0, 0.0];
            this.ctx._transform = this._matrix_multiply(this.ctx._transform, matrix);
        },

        scale: function (sx, sy) {
            var matrix = [sx, 0.0, 0.0, sy, 0.0, 0.0];
            this.ctx._transform = this._matrix_multiply(this.ctx._transform, matrix);
        },

        translate: function (x, y) {
            var matrix = [1.0, 0.0, 0.0, 1.0, x, y];
            this.ctx._transform = this._matrix_multiply(this.ctx._transform, matrix);
        },

        stroke: function () {
            if (this.ctx._clip_path.length > 0) {

                var lines;
                if (window.outIntercept) {
                    lines = window.outIntercept.type === 'group' ? window.outIntercept.stream : window.outIntercept;
                } else {
                    lines = this.pdf.internal.pages[1];
                }
                lines.push("q");

                var origPath = this.path;
                this.path = this.ctx._clip_path;
                this.ctx._clip_path = [];
                this._stroke(true);

                this.ctx._clip_path = this.path;
                this.path = origPath;
                this._stroke(false);

                lines.push("Q");
            } else {
                this._stroke(false);
            }
        },

        _stroke: function (isClip) {
            if (!isClip && this._isStrokeTransparent()) {
                return;
            }

            //TODO opacity

            var moves = [];
            var closed = false;

            var xPath = this.path;

            for (var i = 0; i < xPath.length; i++) {
                var pt = xPath[i];
                switch (pt.type) {
                    case 'mt':
                        moves.push({start: pt, deltas: [], abs: []});
                        break;
                    case 'lt':
                        var delta = [
                            pt.x - xPath[i - 1].x, pt.y - xPath[i - 1].y
                        ];
                        moves[moves.length - 1].deltas.push(delta);
                        moves[moves.length - 1].abs.push(pt);
                        break;
                    case 'bct':
                        var delta = [
                            pt.x1 - xPath[i - 1].x, pt.y1 - xPath[i - 1].y,
                            pt.x2 - xPath[i - 1].x, pt.y2 - xPath[i - 1].y,
                            pt.x - xPath[i - 1].x, pt.y - xPath[i - 1].y
                        ];
                        moves[moves.length - 1].deltas.push(delta);
                        break;
                    case 'qct':
                        // convert to bezier
                        var x1 = xPath[i - 1].x + 2.0 / 3.0 * (pt.x1 - xPath[i - 1].x);
                        var y1 = xPath[i - 1].y + 2.0 / 3.0 * (pt.y1 - xPath[i - 1].y);
                        var x2 = pt.x + 2.0 / 3.0 * (pt.x1 - pt.x);
                        var y2 = pt.y + 2.0 / 3.0 * (pt.y1 - pt.y);
                        var x3 = pt.x;
                        var y3 = pt.y;
                        var delta = [
                            x1 - xPath[i - 1].x, y1 - xPath[i - 1].y,
                            x2 - xPath[i - 1].x, y2 - xPath[i - 1].y,
                            x3 - xPath[i - 1].x, y3 - xPath[i - 1].y
                        ];
                        moves[moves.length - 1].deltas.push(delta);
                        break;
                    case 'arc':
                        moves[moves.length - 1].arc = true;
                        moves[moves.length - 1].abs.push(pt);
                        break;
                    case 'close':
                        closed = true;
                        break;
                }
            }

            for (var i = 0; i < moves.length; i++) {
                var style;
                if (i == moves.length - 1) {
                    style = 's';
                } else {
                    style = null;
                }
                if (moves[i].arc) {
                    var arcs = moves[i].abs;
                    for (var ii = 0; ii < arcs.length; ii++) {
                        var arc = arcs[ii];
                        var start = arc.startAngle * 360 / (2 * Math.PI);
                        var end = arc.endAngle * 360 / (2 * Math.PI);
                        var x = arc.x;
                        var y = arc.y;
                        this.internal.arc2(this, x, y, arc.radius, start, end, arc.anticlockwise, style, isClip);
                    }
                } else {
                    var x = moves[i].start.x;
                    var y = moves[i].start.y;
                    if (!isClip) {
                        this.pdf.lines(moves[i].deltas, x, y, null, style);
                    } else {
                        this.pdf.lines(moves[i].deltas, x, y, null, null);
                        this.pdf.clip_fixed();
                    }
                }
            }
        },

        _isFillTransparent: function () {
            return this.ctx._isFillTransparent || this.globalAlpha == 0;
        },

        _isStrokeTransparent: function () {
            return this.ctx._isStrokeTransparent || this.globalAlpha == 0;
        },

        fill: function (fillRule) { //evenodd or nonzero (default)
            if (this.ctx._clip_path.length > 0) {

                var lines;
                if (window.outIntercept) {
                    lines = window.outIntercept.type === 'group' ? window.outIntercept.stream : window.outIntercept;
                } else {
                    lines = this.pdf.internal.pages[1];
                }
                lines.push("q");

                var origPath = this.path;
                this.path = this.ctx._clip_path;
                this.ctx._clip_path = [];
                this._fill(fillRule, true);

                this.ctx._clip_path = this.path;
                this.path = origPath;
                this._fill(fillRule, false);

                lines.push('Q');
            } else {
                this._fill(fillRule, false);
            }
        },

        _fill: function (fillRule, isClip) {
            if (this._isFillTransparent()) {
                return;
            }
            var v2Support = typeof this.pdf.internal.newObject2 === 'function';

            var lines;
            if (window.outIntercept) {
                lines = window.outIntercept.type === 'group' ? window.outIntercept.stream : window.outIntercept;
            } else {
                lines = this.pdf.internal.pages[1];
            }

            // if (this.ctx._clip_path.length > 0) {
            //     lines.push('q');
            //     var oldPath = this.path;
            //     this.path = this.ctx._clip_path;
            //     this.ctx._clip_path = [];
            //     this._fill(fillRule, true);
            //     this.ctx._clip_path = this.path;
            //     this.path = oldPath;
            // }

            var moves = [];
            var outInterceptOld = window.outIntercept;

            if (v2Support) {
                // Blend and Mask
                switch (this.ctx.globalCompositeOperation) {
                    case 'normal':
                    case 'source-over':
                        break;
                    case 'destination-in':
                    case 'destination-out':
                        //TODO this need to be added to the current group or page
                        // define a mask stream
                        var obj = this.pdf.internal.newStreamObject();

                        // define a mask state
                        var obj2 = this.pdf.internal.newObject2();
                        obj2.push('<</Type /ExtGState');
                        obj2.push('/SMask <</S /Alpha /G ' + obj.objId + ' 0 R>>'); // /S /Luminosity will need to define color space
                        obj2.push('>>');

                        // add mask to page resources
                        var gsName = 'MASK' + obj2.objId;
                        this.pdf.internal.addGraphicsState(gsName, obj2.objId);

                        var instruction = '/' + gsName + ' gs';
                        // add mask to page, group, or stream
                        lines.splice(0, 0, 'q');
                        lines.splice(1, 0, instruction);
                        lines.push('Q');

                        window.outIntercept = obj;
                        break;
                    default:
                        var dictionaryEntry = '/' + this.pdf.internal.blendModeMap[this.ctx.globalCompositeOperation.toUpperCase()];
                        if (dictionaryEntry) {
                            this.pdf.internal.out(dictionaryEntry + ' gs');
                        }
                        break;
                }
            }

            var alpha = this.ctx.globalAlpha;
            if (this.ctx._fillOpacity < 1) {
                // TODO combine this with global opacity
                alpha = this.ctx._fillOpacity;
            }

            //TODO check for an opacity graphics state that was already created
            //TODO do not set opacity if current value is already active
            if (v2Support) {
                var objOpac = this.pdf.internal.newObject2();
                objOpac.push('<</Type /ExtGState');
                //objOpac.push(this.ctx.globalAlpha + " CA"); // Stroke
                //objOpac.push(this.ctx.globalAlpha + " ca"); // Not Stroke
                objOpac.push('/CA ' + alpha); // Stroke
                objOpac.push('/ca ' + alpha); // Not Stroke
                objOpac.push('>>');
                var gsName = 'GS_O_' + objOpac.objId;
                this.pdf.internal.addGraphicsState(gsName, objOpac.objId);
                this.pdf.internal.out('/' + gsName + ' gs');
            }

            var xPath = this.path;

            for (var i = 0; i < xPath.length; i++) {
                var pt = xPath[i];
                switch (pt.type) {
                    case 'mt':
                        moves.push({start: pt, deltas: [], abs: []});
                        break;
                    case 'lt':
                        var delta = [
                            pt.x - xPath[i - 1].x, pt.y - xPath[i - 1].y
                        ];
                        moves[moves.length - 1].deltas.push(delta);
                        moves[moves.length - 1].abs.push(pt);
                        break;
                    case 'bct':
                        var delta = [
                            pt.x1 - xPath[i - 1].x, pt.y1 - xPath[i - 1].y,
                            pt.x2 - xPath[i - 1].x, pt.y2 - xPath[i - 1].y,
                            pt.x - xPath[i - 1].x, pt.y - xPath[i - 1].y
                        ];
                        moves[moves.length - 1].deltas.push(delta);
                        break;
                    case 'qct':
                        // convert to bezier
                        var x1 = xPath[i - 1].x + 2.0 / 3.0 * (pt.x1 - xPath[i - 1].x);
                        var y1 = xPath[i - 1].y + 2.0 / 3.0 * (pt.y1 - xPath[i - 1].y);
                        var x2 = pt.x + 2.0 / 3.0 * (pt.x1 - pt.x);
                        var y2 = pt.y + 2.0 / 3.0 * (pt.y1 - pt.y);
                        var x3 = pt.x;
                        var y3 = pt.y;
                        var delta = [
                            x1 - xPath[i - 1].x, y1 - xPath[i - 1].y,
                            x2 - xPath[i - 1].x, y2 - xPath[i - 1].y,
                            x3 - xPath[i - 1].x, y3 - xPath[i - 1].y
                        ];
                        moves[moves.length - 1].deltas.push(delta);
                        break;
                    case 'arc':
                        //TODO this was hack to avoid out of bounds issue
                        if (moves.length == 0) {
                            moves.push({start: {x: 0, y: 0}, deltas: [], abs: []});
                        }
                        moves[moves.length - 1].arc = true;
                        moves[moves.length - 1].abs.push(pt);
                        break;
                    case 'close':
                        //moves[moves.length - 1].deltas.push('close');
                        break;
                }
            }

            for (var i = 0; i < moves.length; i++) {
                var style;
                if (i == moves.length - 1) {
                    style = 'f';
                    if (fillRule === 'evenodd') {
                        style += '*';
                    }
                } else {
                    style = null;
                }

                if (moves[i].arc) {
                    var arcs = moves[i].abs;
                    for (var ii = 0; ii < arcs.length; ii++) {
                        var arc = arcs[ii];
                        //TODO lines deltas were getting in here
                        if (typeof arc.startAngle !== 'undefined') {
                            var start = arc.startAngle * 360 / (2 * Math.PI);
                            var end = arc.endAngle * 360 / (2 * Math.PI);
                            // Add the current position (last move to)
                            //var x = moves[i].start.x + arc.x;
                            //var y = moves[i].start.y + arc.y;
                            var x = arc.x;
                            var y = arc.y;
                            if (ii == 0) {
                                this.internal.move2(this, x, y);
                            }
                            this.internal.arc2(this, x, y, arc.radius, start, end, arc.anticlockwise, null, isClip);
                        } else {
                            this.internal.line2(c2d, arc.x, arc.y);
                        }
                    }

                    // extra move bug causing close to resolve to wrong point
                    var x = moves[i].start.x;
                    var y = moves[i].start.y;
                    this.internal.line2(c2d, x, y);

                    this.pdf.internal.out('h');
                    this.pdf.internal.out('f');
                }
                else {
                    var x = moves[i].start.x;
                    var y = moves[i].start.y;
                    if (!isClip) {
                        this.pdf.lines(moves[i].deltas, x, y, null, style);
                    } else {
                        this.pdf.lines(moves[i].deltas, x, y, null, null);
                        this.pdf.clip_fixed();
                    }
                }
            }

            window.outIntercept = outInterceptOld;

            // if (this.ctx._clip_path.length > 0) {
            //     lines.push('Q');
            // }
        },

        pushMask: function () {
            var v2Support = typeof this.pdf.internal.newObject2 === 'function';

            if (!v2Support) {
                console.log('jsPDF v2 not enabled')
                return;
            }

            // define a mask stream
            var obj = this.pdf.internal.newStreamObject();

            // define a mask state
            var obj2 = this.pdf.internal.newObject2();
            obj2.push('<</Type /ExtGState');
            obj2.push('/SMask <</S /Alpha /G ' + obj.objId + ' 0 R>>'); // /S /Luminosity will need to define color space
            obj2.push('>>');

            // add mask to page resources
            var gsName = 'MASK' + obj2.objId;
            this.pdf.internal.addGraphicsState(gsName, obj2.objId);

            var instruction = '/' + gsName + ' gs';
            this.pdf.internal.out(instruction);
        },

        clip: function () {
            //TODO do we reset the path, or just copy it?
            if (this.ctx._clip_path.length > 0) {
                for (var i = 0; i < this.path.length; i++) {
                    this.ctx._clip_path.push(this.path[i]);
                }
            } else {
                this.ctx._clip_path = this.path;
            }
            this.path = [];
        },

        measureText: function (text) {
            var pdf = this.pdf;
            return {
                getWidth: function () {
                    var fontSize = pdf.internal.getFontSize();
                    var txtWidth = pdf.getStringUnitWidth(text) * fontSize / pdf.internal.scaleFactor;
                    return txtWidth;
                },

                get width() {
                    return this.getWidth(text);
                }
            }
        },
        _getBaseline: function (y) {
            var height = parseInt(this.pdf.internal.getFontSize());
            // TODO Get descent from font descriptor
            var descent = height * .25;
            switch (this.ctx.textBaseline) {
                case 'bottom':
                    return y - descent;
                case 'top':
                    return y + height;
                case 'hanging':
                    return y + height - descent;
                case 'middle':
                    return y + height / 2 - descent;
                case 'ideographic':
                    // TODO not implemented
                    return y;
                case 'alphabetic':
                default:
                    return y;
            }
        }
    }
    ;

    var c2d = jsPDFAPI.context2d;

    // accessor methods
    Object.defineProperty(c2d, 'fillStyle', {
        set: function (value) {
            this.setFillStyle(value);
        },
        get: function () {
            return this.ctx.fillStyle;
        }
    });
    Object.defineProperty(c2d, 'strokeStyle', {
        set: function (value) {
            this.setStrokeStyle(value);
        },
        get: function () {
            return this.ctx.strokeStyle;
        }
    });
    Object.defineProperty(c2d, 'lineWidth', {
        set: function (value) {
            this.setLineWidth(value);
        },
        get: function () {
            return this.ctx.lineWidth;
        }
    });
    Object.defineProperty(c2d, 'lineCap', {
        set: function (val) {
            this.setLineCap(val);
        },
        get: function () {
            return this.ctx.lineCap;
        }
    });
    Object.defineProperty(c2d, 'lineJoin', {
        set: function (val) {
            this.setLineJoin(val);
        },
        get: function () {
            return this.ctx.lineJoin;
        }
    });
    Object.defineProperty(c2d, 'miterLimit', {
        set: function (val) {
            this.ctx.miterLimit = val;
        },
        get: function () {
            return this.ctx.miterLimit;
        }
    });
    Object.defineProperty(c2d, 'textBaseline', {
        set: function (value) {
            this.setTextBaseline(value);
        },
        get: function () {
            return this.getTextBaseline();
        }
    });
    Object.defineProperty(c2d, 'textAlign', {
        set: function (value) {
            this.setTextAlign(value);
        },
        get: function () {
            return this.getTextAlign();
        }
    });
    Object.defineProperty(c2d, 'font', {
        set: function (value) {
            this.setFont(value);
        },
        get: function () {
            return this.ctx.font;
        }
    });
    Object.defineProperty(c2d, 'globalCompositeOperation', {
        set: function (value) {
            this.ctx.globalCompositeOperation = value;
        },
        get: function () {
            return this.ctx.globalCompositeOperation;
        }
    });
    Object.defineProperty(c2d, 'globalAlpha', {
        set: function (value) {
            this.ctx.globalAlpha = value;
        },
        get: function () {
            return this.ctx.globalAlpha;
        }
    });
    // Not HTML API
    Object.defineProperty(c2d, 'ignoreClearRect', {
        set: function (value) {
            this.ctx.ignoreClearRect = value;
        },
        get: function () {
            return this.ctx.ignoreClearRect;
        }
    });
    // End Not HTML API

    c2d.internal = {};

    c2d.internal.rxRgb = /rgb\s*\(\s*(\d+)\s*,\s*(\d+)\s*,\s*(\d+)\s*\)/;
    c2d.internal.rxRgba = /rgba\s*\(\s*(\d+)\s*,\s*(\d+)\s*,\s*(\d+)\s*,\s*([\d\.]+)\s*\)/;
    c2d.internal.rxTransparent = /transparent|rgba\s*\(\s*(\d+)\s*,\s*(\d+)\s*,\s*(\d+)\s*,\s*0+\s*\)/;

    // http://hansmuller-flex.blogspot.com/2011/10/more-about-approximating-circular-arcs.html
    c2d.internal.arc = function (c2d, xc, yc, r, a1, a2, anticlockwise, style) {
        var includeMove = true;

        var k = this.pdf.internal.scaleFactor;
        var pageHeight = this.pdf.internal.pageSize.height;
        var f2 = this.pdf.internal.f2;

        var a1r = a1 * (Math.PI / 180);
        var a2r = a2 * (Math.PI / 180);
        var curves = this.createArc(r, a1r, a2r, anticlockwise);
        var pathData = null;

        for (var i = 0; i < curves.length; i++) {
            var curve = curves[i];
            if (includeMove && i == 0) {
                this.pdf.internal.out([
                    f2((curve.x1 + xc) * k), f2((pageHeight - (curve.y1 + yc)) * k), 'm', f2((curve.x2 + xc) * k), f2((pageHeight - (curve.y2 + yc)) * k), f2((curve.x3 + xc) * k), f2((pageHeight - (curve.y3 + yc)) * k), f2((curve.x4 + xc) * k), f2((pageHeight - (curve.y4 + yc)) * k), 'c'
                ].join(' '));

            } else {
                this.pdf.internal.out([
                    f2((curve.x2 + xc) * k), f2((pageHeight - (curve.y2 + yc)) * k), f2((curve.x3 + xc) * k), f2((pageHeight - (curve.y3 + yc)) * k), f2((curve.x4 + xc) * k), f2((pageHeight - (curve.y4 + yc)) * k), 'c'
                ].join(' '));
            }

            //c2d._lastPoint = {x: curve.x1 + xc, y: curve.y1 + yc};
            c2d._lastPoint = {x: xc, y: yc};
            // f2((curve.x1 + xc) * k), f2((pageHeight - (curve.y1 + yc)) * k), 'm', f2((curve.x2 + xc) * k), f2((pageHeight - (curve.y2 + yc)) * k), f2((curve.x3 + xc) * k), f2((pageHeight - (curve.y3 + yc)) * k), f2((curve.x4 + xc) * k), f2((pageHeight - (curve.y4 + yc)) * k), 'c'
        }

        if (style !== null) {
            this.pdf.internal.out(this.pdf.internal.getStyle(style));
        }
    };

    /**
     *
     * @param x Edge point X
     * @param y Edge point Y
     * @param r Radius
     * @param a1 start angle
     * @param a2 end angle
     * @param anticlockwise
     * @param style
     * @param isClip
     */
    c2d.internal.arc2 = function (c2d, x, y, r, a1, a2, anticlockwise, style, isClip) {
        // we need to convert from cartesian to polar here methinks.
        var centerX = x;// + r;
        var centerY = y;

        if (false) {
            var phi = (a2 - a1);
            var start = {
                x: r,
                y: 0
            };

            var pt1 = {
                x: r,
                y: r * 4 / 3 * Math.tan(phi / 4)
            };

            var pt2 = {
                x: r * ( Math.cos(phi) + 4 / 3 * Math.tan(phi / 4) * Math.sin(phi) ),
                y: r * ( Math.sin(phi) - 4 / 3 * Math.tan(phi / 4) * Math.cos(phi) )
            };

            var end = {
                x: r * Math.cos(phi),
                y: r * Math.sin(phi)
            };

            var matrix = [Math.cos(a1), Math.sin(a1), -Math.sin(a1), Math.cos(a1), x, y];
            start = c2d._matrix_map_point_obj(matrix, start);
            pt1 = c2d._matrix_map_point_obj(matrix, pt1);
            pt2 = c2d._matrix_map_point_obj(matrix, pt2);
            end = c2d._matrix_map_point_obj(matrix, end);

            var k = this.pdf.internal.scaleFactor;
            var pageHeight = this.pdf.internal.pageSize.height;
            var f2 = this.pdf.internal.f2;
            this.pdf.internal.out([
                f2((start.x) * k), f2((pageHeight - (start.y)) * k), 'm', f2((pt1.x) * k), f2((pageHeight - (pt1.y)) * k), f2((pt2.x) * k), f2((pageHeight - (pt2.y)) * k), f2((end.x) * k), f2((pageHeight - (end.y)) * k), 'c'
            ].join(' '));
            //this.pdf.internal.out('f');
            c2d._lastPoint = end;
            return;
        }

        if (!isClip) {
            this.arc(c2d, centerX, centerY, r, a1, a2, anticlockwise, style);
        } else {
            this.arc(c2d, centerX, centerY, r, a1, a2, anticlockwise, null);
            this.pdf.clip_fixed();
        }
    };

    c2d.internal.move2 = function (c2d, x, y) {
        var k = this.pdf.internal.scaleFactor;
        var pageHeight = this.pdf.internal.pageSize.height;
        var f2 = this.pdf.internal.f2;

        this.pdf.internal.out([
            f2((x) * k), f2((pageHeight - (y)) * k), 'm'
        ].join(' '));
        c2d._lastPoint = {x: x, y: y};
    };

    c2d.internal.line2 = function (c2d, dx, dy) {
        var k = this.pdf.internal.scaleFactor;
        var pageHeight = this.pdf.internal.pageSize.height;
        var f2 = this.pdf.internal.f2;

        //var pt = {x: c2d._lastPoint.x + dx, y: c2d._lastPoint.y + dy};
        var pt = {x: dx, y: dy};

        this.pdf.internal.out([
            f2((pt.x) * k), f2((pageHeight - (pt.y)) * k), 'l'
        ].join(' '));
        //this.pdf.internal.out('f');
        c2d._lastPoint = pt;

    };

    /**
     * Return a array of objects that represent bezier curves which approximate the circular arc centered at the origin, from startAngle to endAngle (radians) with the specified radius.
     *
     * Each bezier curve is an object with four points, where x1,y1 and x4,y4 are the arc's end points and x2,y2 and x3,y3 are the cubic bezier's control points.
     */

    c2d.internal.createArc = function (radius, startAngle, endAngle, anticlockwise) {

        var EPSILON = 0.00001; // Roughly 1/1000th of a degree, see below

        // normalize startAngle, endAngle to [-2PI, 2PI]
        var twoPI = Math.PI * 2;
        var startAngleN = startAngle;
        if (startAngleN < twoPI || startAngleN > twoPI) {
            startAngleN = startAngleN % twoPI;
        }
        var endAngleN = endAngle;
        if (endAngleN < twoPI || endAngleN > twoPI) {
            endAngleN = endAngleN % twoPI;
        }

        // Compute the sequence of arc curves, up to PI/2 at a time.
        // Total arc angle is less than 2PI.
        var curves = [];
        var piOverTwo = Math.PI / 2.0;
        // var sgn = (startAngle < endAngle) ? +1 : -1; // clockwise or counterclockwise
        var sgn = anticlockwise ? -1 : +1;

        var a1 = startAngle;
        for (var totalAngle = Math.min(twoPI, Math.abs(endAngleN - startAngleN)); totalAngle > EPSILON;) {
            var a2 = a1 + sgn * Math.min(totalAngle, piOverTwo);
            curves.push(this.createSmallArc(radius, a1, a2));
            totalAngle -= Math.abs(a2 - a1);
            a1 = a2;
        }

        return curves;
    };

    /**
     * Cubic bezier approximation of a circular arc centered at the origin, from (radians) a1 to a2, where a2-a1 < pi/2. The arc's radius is r.
     *
     * Returns an object with four points, where x1,y1 and x4,y4 are the arc's end points and x2,y2 and x3,y3 are the cubic bezier's control points.
     *
     * This algorithm is based on the approach described in: A. Riškus, "Approximation of a Cubic Bezier Curve by Circular Arcs and Vice Versa," Information Technology and Control, 35(4), 2006 pp. 371-378.
     */

    c2d.internal.createSmallArc = function (r, a1, a2) {
        // Compute all four points for an arc that subtends the same total angle
        // but is centered on the X-axis

        var a = (a2 - a1) / 2.0;

        var x4 = r * Math.cos(a);
        var y4 = r * Math.sin(a);
        var x1 = x4;
        var y1 = -y4;

        var q1 = x1 * x1 + y1 * y1;
        var q2 = q1 + x1 * x4 + y1 * y4;
        var k2 = 4 / 3 * (Math.sqrt(2 * q1 * q2) - q2) / (x1 * y4 - y1 * x4);

        var x2 = x1 - k2 * y1;
        var y2 = y1 + k2 * x1;
        var x3 = x2;
        var y3 = -y2;

        // Find the arc points' actual locations by computing x1,y1 and x4,y4
        // and rotating the control points by a + a1

        var ar = a + a1;
        var cos_ar = Math.cos(ar);
        var sin_ar = Math.sin(ar);

        return {
            x1: r * Math.cos(a1),
            y1: r * Math.sin(a1),
            x2: x2 * cos_ar - y2 * sin_ar,
            y2: x2 * sin_ar + y2 * cos_ar,
            x3: x3 * cos_ar - y3 * sin_ar,
            y3: x3 * sin_ar + y3 * cos_ar,
            x4: r * Math.cos(a2),
            y4: r * Math.sin(a2)
        };
    }

    function context() {
        this._isStrokeTransparent = false;
        this._strokeOpacity = 1;
        this.strokeStyle = '#000000';
        this.fillStyle = '#000000';
        this._isFillTransparent = false;
        this._fillOpacity = 1;
        this.font = "12pt times";
        this.textBaseline = 'alphabetic'; // top,bottom,middle,ideographic,alphabetic,hanging
        this.textAlign = 'start';
        this.lineWidth = 1;
        this.lineJoin = 'miter'; // round, bevel, miter
        this.lineCap = 'butt'; // butt, round, square
        this._transform = [1, 0, 0, 1, 0, 0]; // sx, shy, shx, sy, tx, ty
        this.globalCompositeOperation = 'normal';
        this.globalAlpha = 1.0;
        this._clip_path = [];
        // TODO miter limit //default 10

        // Not HTML API
        this.ignoreClearRect = false;

        this.copy = function (ctx) {
            this._isStrokeTransparent = ctx._isStrokeTransparent;
            this._strokeOpacity = ctx._strokeOpacity;
            this.strokeStyle = ctx.strokeStyle;
            this._isFillTransparent = ctx._isFillTransparent;
            this._fillOpacity = ctx._fillOpacity;
            this.fillStyle = ctx.fillStyle;
            this.font = ctx.font;
            this.lineWidth = ctx.lineWidth;
            this.lineJoin = ctx.lineJoin;
            this.lineCap = ctx.lineCap;
            this.textBaseline = ctx.textBaseline;
            this.textAlign = ctx.textAlign;
            this._fontSize = ctx._fontSize;
            this._transform = ctx._transform.slice(0);
            this.globalCompositeOperation = ctx.globalCompositeOperation;
            this.globalAlpha = ctx.globalAlpha;
            this._clip_path = ctx._clip_path.slice(0); //TODO deep copy?

            // Not HTML API
            this.ignoreClearRect = ctx.ignoreClearRect;
        };
    }

    return this;
})(jsPDF.API);
```

  

Documentation generated by JSDoc 3.4.2 on Sun Oct 09 2016 11:08:27 GMT+0100 (BST) using the docdash theme.
